# Ecological Impacts of Deep-Sea Mining Waste on Marine Algae and Copepod *Tigriopus californicus*

**DOI:** 10.1021/acs.est.5c06113

**Published:** 2025-09-18

**Authors:** Catherine Thomson, Alastair J. M. Lough, Jean Moorkens, Te Liu, Shelby A. Gunnells, Jessica N. Fitzsimmons, Zvi Steiner, Ann G. Dunlea, Clare Woulds, William B. Homoky, Mengjiao Wang, Qiao-Guo Tan, Fengjie Liu

**Affiliations:** † Centre for Environmental Policy, 4615 Imperial College London, Exhibition Road, London, SW7 2AZ, U.K.; ‡ Faculty of Environment, University of Leeds, Leeds, LS2 9JT, U.K.; § Department of Life Sciences, 152935 Imperial College London, Silwood Park, Buckhurst Rd, Berks SL5 7PY, U.K.; ∥ School of Ocean and Earth Science, 7423 University of Southampton, Southampton SO14 3ZH, U.K.; ⊥ Department of Oceanography, 14736 Texas A&M University, College Station, Texas 77843, United States; # Fredy and Nadine Herrmann Institute of Earth Science, 28402 The Hebrew University of Jerusalem, Givat Ram, Jerusalem, 9190401, Israel; ∇ Department of Marine Chemistry and Geochemistry, Woods Hole Oceanographic Institution, Woods Hole, Massachusetts 02543, United States; ○ Greenpeace Research Laboratories, School of Biosciences, 196740 University of Exeter, Exeter EX4 4RN, U.K.; ◆ Fujian Provincial Key Laboratory for Coastal Ecology and Environmental Studies, State Key Lab of Marine Environmental Science, College of the Environment and Ecology, 12466 Xiamen University, Xiamen, Fujian 361102, China; ¶ Grantham Institute - Climate Change and the Environment, Imperial College London, Exhibition Road, London SW7 2AZ, U.K.

**Keywords:** deep-sea mining, Clarion−Clipperton zone, ecological impacts, environmental risk assessment, phytoplankton, zooplankton, metal toxicity, suspended sediments

## Abstract

With growing global interest in critical metals, large-scale operations are increasingly proposed by some for mining across deep-sea ecosystems, raising concerns about environmental impacts on and beyond the seafloor. Discharges of mining-derived sediments and effluent into the pelagic zone can spread contamination beyond benthic environments, particularly via diel vertically migrating species, affecting epipelagic and mesopelagic communities. This study investigates the effects of sediments from the Clarion–Clipperton zone (CCZ) on representative phytoplankton and zooplankton under laboratory conditions, focusing on nutrient availability, metal toxicity, and reproductive impacts. Sediment particles stimulated the growth of nitrogen- or metal-limited diatoms (*Thalassiosira weissflogii*, *Phaeodactylum tricornutum*, *Skeletonema costatum*), coccolithophores (*Emiliania huxleyi*), and cyanobacteria (*Synechococcus sp.*) by releasing nutrients (N, Co, Cu, Fe, Mn, Ni, and Zn). However, a reduced growth of the diatom *T. weissflogii* in metal-replete seawater and a limited response of cyanobacteria *Synechococcus* were observed, likely due to metal toxicity. The marine copepod *Tigriopus californicus* exhibited dose-dependent reductions in growth and reproduction to the CCZ sediment (2–50 mg L^–1^) and significant reductions in mating success, pregnancy rates, and offspring viability were also observed following exposure to the sediment from the North Pacific abyss. While the test species primarily inhabit surface waters, they were selected as established models to elucidate mechanistic responses to deep-sea sediment exposure. These findings provide one of the first assessments of ecological vulnerabilities to deep-sea mining waste discharges that are broadly relevant across pelagic ecosystems and could inform regulatory decisions by the International Seabed Authority or any individual nations seeking to mine the deep-sea beyond national jurisdiction.

## Graphical abstract

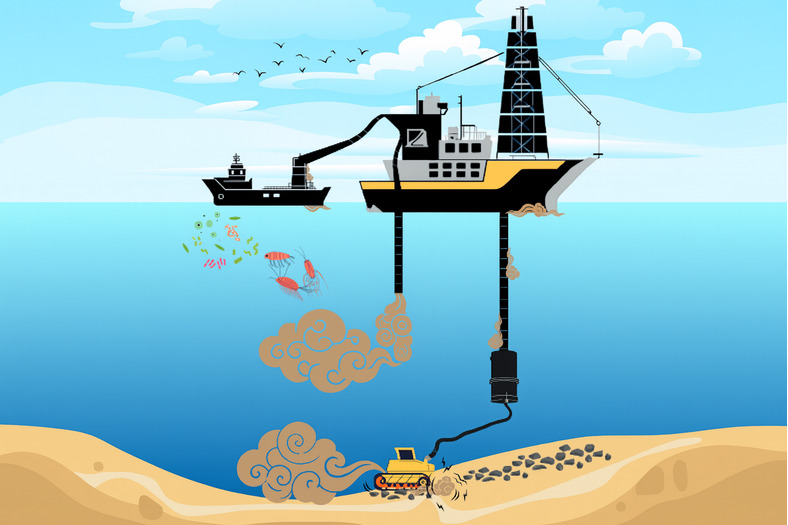

## Introduction

Metals such as copper (Cu), cobalt (Co), manganese (Mn), and nickel (Ni) play a crucial role in renewable energy technologies and the transition to a low-carbon economy. These metals are essential components in advanced batteries, solar panels, wind turbines, and other clean energy innovations that are key to reducing global reliance on fossil fuels. The accelerating transition toward green energy has yielded divergent projections for global critical metals demand, varying substantially based on assumptions of different transition pathways, including one scenario suggesting a doubling by 2060,^1^ far surpassing current production levels from traditional terrestrial mining. However, land-based reserves are increasingly constrained by environmental concerns, geopolitical factors, and declining ore grades, driving interest in alternative sources. An alternative to terrestrial sources proposed by some is deep-sea mining, particularly in the Clarion–Clipperton zone, which holds over 21 billion metric tons of metal-rich deposits known as polymetallic nodules.^2^ As deep-sea mining technologies advance, the Clarion–Clipperton zone and other abyssal plains are being explored as potential sources to meet the growing demand for these strategic metals and support the global transition to sustainable energy.^3^ The International Seabed Authority (ISA) is responsible for regulating deep-sea mining in international waters under the UN Convention on the Law of the Sea (UNCLOS) and is currently still in the process of developing regulations. Some nongovernmental organizations and ISA member states are calling for a moratorium on deep-sea mining, often citing the limited amount of data available to assess impact as part of their rationale.

In almost all proposed operations of deep-sea mining, extracted deposits are transported from the seafloor via a vertical riser system to a surface support vessel, where onboard processing occurs. The resulting wastewater composed of deep-sea sediments and nodule fragments is subsequently discharged into the ocean, though the precise depth of discharge remains uncertain.^4^ These discharges inevitably introduce contaminants into seawater, potentially impacting biological communities.^5^ A recent deep-sea mining test in the Clarion–Clipperton zone resulted in the accidental release of a small amount of sediment and nodule fragments into the surface waters of the Pacific Ocean,^6^ underscoring the need to integrate surface ecosystems in evaluation of deep-sea mining impacts. However, research on the ecological impact of deep-sea mining in upper ocean layers is extremely limited, with most existing studies only focusing on deep-sea ecosystems.^7–12^

Metals from marine sediments are released in both dissolved and particulate forms.^13^ A recent laboratory study found that deep-sea mining waste releases Mn, Ni, Cu, Co, Cd, and Pb into ambient waters, with Co and Cu being the most enriched within the plume—reaching concentrations up to ∼15 times higher than background seawater levels.^14^ If this waste were introduced into the surface ocean, the elevated concentrations and bioavailability of nutrients including metals (e.g., Fe) could stimulate phytoplankton growth, particularly in regions where primary productivity is limited by N and/or metal availability.^15^ However, an onboard incubation experiment in the western Pacific revealed that deep-sea mining slurry and sediments can stimulate, inhibit, or have no effect on *in situ* phytoplankton communities, with the underlying reasons for these contrasting effects remaining unclear.^16^ Additionally, the dewatering process of deep-sea mining will generate particulate plumes, with fine suspended particles persisting for weeks to months depending on the depth of discharge.^17^ These particles are likely to be ingested by many zooplankton species, particularly filter feeders. Elevated concentrations of suspended particles impair zooplankton feeding efficiency, growth, and reproduction.^18,19^ Currently, there is limited knowledge of the potential impacts of deep-sea mining waste discharge and accidental leaks on both phytoplankton and zooplankton. Marine algae and copepods play vital ecological roles in global aquatic food webs. As primary producers and key grazers, respectively, their growth and reproduction are fundamental to sustaining marine ecosystem functions and services.^20^

In this study, we use slurry from an incidental deep-sea mining surface spill^6^ and surface sediments from the Clarion–Clipperton zone in the North Pacific for a series of laboratory experiments. We conducted the experiments under trace metal-clean conditions to investigate the effects of these materials on the growth of model phytoplankton species, including diatoms, coccolithophores, and cyanobacteria, as well as the growth and reproduction of model zooplankton copepods. Particularly, we examined the bioavailability of Co, Cu, Fe, Mn, Ni, and Zn associated with sediment particles by excluding each of these metals from the algal growth media. Additionally, we used surface water from the Pacific Ocean to assess the growth response of diatoms to suspended sediment particles at environmentally relevant concentrations (0–50 mg L^–1^).^21^ This study provides one of the first assessments of the ecological impact of deep-sea mining waste discharge on epi- and mesopelagic zones, offering valuable insights to inform future deep-sea mining discharge operations and industry regulations.

## Materials and Methods

### Collection of Deep-Sea Mining Slurry, Sediments, and Surface Seawater

During a deep-sea mining test activity in the fall of 2022 in the NORI-D lease region (10.32°N, 117.18°W, Figure S1), some uncontained mining slurry from the onboard processing system spilled over the sides of the surface production vessel (M/V *Hidden Gem*) into the ocean.^6^ During the short time the slurry was falling overboard, this material was captured into 500 mL LDPE bottles. Samples were kept frozen at −20 °C. Onshore, samples were dethawed, vigorously shaken, and filtered through acid-washed 47 mm 0.2 μm poly­(ether sulfone) (Supor) filters. Filtered macronutrient samples were collected and analyzed at ODF at Scripps Institution of Oceanography. Dissolved metal samples were collected into acid-washed 30 mL LDPE bottles and acidified to 0.024 M HCl. Dissolved metal concentrations (Table S1) were measured using an offline automated SeaFAST preconcentration step^22,23^ paired with inductively coupled plasma mass spectrometry (ICP-MS) analysis on an Element XR at Texas A&M University.

Sediments were collected using a multicorer (*Oktopus*) from the NORI-D claim area in the eastern Clarion–Clipperton zone (10°50′24″N, 116°09′00″W, site SPR41, Figure S1). Samples were collected in a temperature-controlled lab set to a temperature similar to that of the deep-sea. Core samples were frozen at −20 °C and freeze-dried on shore. Surface sediment (0–0.5 cm) was used for the bioassay experiments as this is the material most likely to be captured by a mining vehicle along with the nodules. Total organic carbon (TOC) and total nitrogen (TN) concentrations were low relative to other settings (0.65 wt % TOC and 0.15 wt % TN) which is typical of deep-sea sediments. Metal concentrations of Co, Cu, Fe, Mn, Ni, and Zn were 0.009, 0.06, 4, 1, 0.04, 0.02 wt %, respectively (Table S1). For grain size analysis particles were binned into <0.001, 0.001–2, 2–6.3, 6.3–20, 20–63, 63–112, 112–200, 200–335, 335–630, 630–1120, 1120–2000 μm size ranges. All particles were <63 μm in size with the volume of particles falling into the first five bins 16.9, 30.9, 31.8, 20.3 and 0.13%, respectively. A sequential extraction was performed to determine the predominant highly reactive Fe mineral phases.^24^ Reactive Fe was predominantly present as Fe oxyhydoxides (Fe_ox_ = 1.14 wt %) with a less amount present as mixed ferrous-ferric oxide phases (Fe_mag_ = 0.25 wt %) and associated with carbonate minerals (Fe_carb_ = 0.19 wt %) leaving the majority of Fe (2.42 wt %) present as less reactive mineral phases (e.g., silicates). Porosity of the wet sediment (f) was 0.80.

Surface sediments (0–1 cm layer) from short cores collected at the North Pacific abyssal plain (49°50.4′N, 149°37.7′W, Figure S1) during the CDisK-IV cruise aboard R/V *Kilo Moana* in August 2017 were also used for the bioassays with marine copepods. The total metal content in the sediment was as follows: 6.99% Al, 27 mg Co kg^–1^, 25 mg Cr kg^–1^, 125 mg Cu kg^–1^, 5.45% Fe, 0.16% Mn, 1 mg Mo kg^–1^, 22 mg Ni kg^–1^, 14 mg Pb kg^–1^, and 95 mg Zn kg^–1^ on a dry weight basis (Table S1). Further details on the sediment’s chemical composition, storage and processing can be found in a previous study.^25^

Surface seawater from the Pacific Ocean was collected for a part of algal bioassays, considering that metal bioavailability depends on water chemistry (e.g., metal ligand types and concentrations).^26^ During the GEOTRACES GP21 expedition aboard the German research vessel *SONNE* (SO289), seawater was sampled at ∼3 m depth in the South Pacific gyre (26°10′12″S, 167°57′00″W) using a trace metal-clean underway tow-fish system. The water was filtered through 0.22 μm membrane filters and contained low concentrations of inorganic nitrogen (5 nM), phosphate (6 nM), dissolved Fe (0.11 nM), Mn (0.87 nM), Zn (0.18 nM), and Co (0.015 nM). Additional seawater chemical properties are reported in a previous study.^27,28^

### Preparation of Exposure Medium

To prepare exposure media with target concentrations of suspended sediment particles, briefly (1) 10–200 mg of sediment was added to 1 L artificial/natural seawater, shaken at 158 rpm for 1 h, and left to settle for another 1 h. This allowed large particles to sink, leaving only suspended particles; (2) Suspended particle concentrations were determined by filtering the upper 0.8 L through preweighed 0.22 μm membrane filters, which were dried at room temperature to constant weight; and (3) Final suspended particle concentrations in different bioassays were 2, 5, 6, 10, 20, 30, or 50 mg L^–1^.

The sediment concentrations used in this study fall within the range measured in plumes generated during deep-sea mining trials. For instance, at a distance of 50 m from a deep-sea mining site boundary,^21^ the highest recorded concentration of suspended sediment particles was 264 mg L^–1^ —4 orders of magnitude higher than the natural background level of 0.02–0.03 mg L^–1^. Even at approximately 1,800 m from the impact site, a maximum concentration of 3.9 mg L^–1^ was recorded. While the particle concentrations resulting from deep-sea mining dewatering or spillage remain unknown, they are likely within a similar range.

For the experiments using the slurry collected from the mining spill, slurry solution was diluted 1:1 with artificial seawater for algal exposure experiments. The 1:1 dilution was selected due to the limited volume of slurry collected, and this dilution may not represent widespread environmental conditions representative of discharge from a mining vessel at sea.

### Bioassays with Marine Phytoplankton

A series of bioassays were conducted to investigate the effects of deep-sea mining-derived slurry and sediment particles on marine phytoplankton, with specific aims to a) assess the overall growth response to these materials; and b) examine whether sediment particles release bioavailable nutrients and trace metals essential for algal growth. To achieve these goals, both artificial and natural seawater were used. Artificial seawater was prepared without the addition of major nutrients and trace metals, enabling us to test whether the slurry and sediment particles could serve as a source of these essential elements. In contrast, natural Pacific Ocean surface seawater was used to more closely represent oceanic conditions, including the presence of natural organic ligands that may influence the speciation and bioavailability of metals released from the sediments. Taken together, we were able to explore mechanistic responses under controlled conditions as well as evaluate the potential ecological relevance of deep-sea sediment inputs in natural seawater environments.

The following species were used: diatoms *Thalassiosira weissflogii* CCMP 1336, *Phaeodactylum tricornutum* CCAP 1055/18, *Skeletonema cf. costatum* RCC70; coccolithophore *Emiliania huxleyi* CCMP 373; and cyanobacterium *Synechococcus* sp. CCAP 1479/22. These species are widely distributed in the global ocean and inhabit the epipelagic zone. Although not native to deeper ocean depths where discharges are often proposed, they were chosen for their ecological importance and established use as model organisms in marine ecotoxicology. Their well-characterized responses provide mechanistic insights into nutrient enrichment and metal toxicity, offering a tractable system to assess potential impacts on pelagic communities, including those affected by vertical plume transport or exposure through migrating species.

All species were maintained in artificial seawater ASW supplied with major nutrients, trace metals and vitamins (Table S2) under controlled conditions (23 °C, 60 or 100 μmol m^– 2^ s^– 1^ light intensity, 12- or 14-h light/dark cycle). The medium was prepared following trace metal-clean protocols,^29^ passed through Chelex-100 resins, and filtered (0.2 μm polycarbonate filters, Merck Millipore Ltd.) before adding trace metals and vitamins. Metals were buffered with 20 μM ethylenediaminetetraacetic acid (EDTA).^29^

To prepare for the bioassays, phytoplankton cells were filtered onto 3 μm membrane filters and harvested at the exponential growth phase. To minimize physiological damage, gravity filtration was used for all species except *Synechococcus* sp., which was collected on 0.22 μm filters with a vacuum pump. Cells were rinsed three times with Chelex-100 pretreated artificial seawater (Table S2) and resuspended in the seawater devoid of major nutrients, trace metals, and vitamins before the start of exposure experiments. They were maintained in the seawater for 2–4 days to deplete intracellular nutrient reserves and extracellular bound metals before exposure experiments.

The phytoplankton were introduced at low cell densities into 40 mL sterile culture flasks containing exposure medium with/without suspended sediment particles or slurry. Any potential change in the concentration of dissolved and particulate metals during the exposure period was not monitored in the present study due to the limited volume of the culture flasks. Each treatment had 2–4 replicate flasks. Cultures were maintained under identical growth conditions, and growth (relative fluorescence units, RFU) was monitored using a Trilogy Laboratory Fluorometer (Turner Designs). Background fluorescence from sediment particles was checked, especially at low algal cell densities, via control experiments with the sediment particles alone. The effect of sediment particles was found to be minor relative to the increases in RFU observed from phytoplankton growth.

### Bioassays with Marine Copepods

To assess the potential effects of deep-sea mining sediments on zooplankton growth and reproduction, two exposure experiments were conducted using the marine copepod *T. californicus*, a widely used model species for ecotoxicological studies.^30^ The first experiment examined the overall growth effects of Clarion–Clipperton zone sediment, and the second experiment investigated growth and reproductive effects using sediment from the North Pacific deep-sea abyss.

Copepods were cultured in the artificial seawater at 23 °C, 12-h light/dark cycle and fed Reefphyto premium copepod feed liquid (a 10% brown, 90% green algae mix, 9% dry weight algae of the feed liquid, UK). They were provided with 100 μL of the feed liquid every 4 days, and adult copepods (13–18 days old, ∼1.5 mm in length) were selected for the exposure experiments.

Six experimental treatments were established for each of the two exposure experiments: Four sediment exposure levels: 2, 5, 20, and 50 mg L^–1^ suspended sediment particles, normal feeding; Positive control: 0 mg L^–1^ sediment, normal feeding; and Negative control: 0 mg L^–1^ sediment, no feeding. The exposure media were prepared as described above. Each treatment had four replicate flasks, with 20 copepods per flask. Before exposure, copepods were prestarved for 3 days. During the experimental period, they were removed daily from each experimental flask using a wide-bore pipet and transferred into three replicate observation containers. Under a stereomicroscope, we recorded the total number of copepods, the number of gravid (egg-carrying) females, and copepodites (juvenile stages). In addition, we observed and noted the presence of mating-guarding behavior, characterized by males clasping onto juvenile or adult females—a known reproductive trait in *T. californicus*.^31^ The medium was renewed every 10 days, and culture flasks were cleaned accordingly.

For the algae and copepods bioassays, due to logistical constraints—including a) limited availability of sediment and slurry samples, b) trace metal-clean protocols, as well as c) staggered experiment timelines—some experimental treatments were not conducted across all species or sediment types. For instance, for the slurry and the Pacific Ocean seawater exposure experiment, only one or two species were tested due to the limited volume of slurry and natural seawater samples available; for other experiments using artificial seawater, we expand to several other algae species to examine broader taxonomic responses. While not all species were included in every assay, each experiment was internally consistent and strategically designed to maximize mechanistic insight while capturing key aspects of phytoplankton taxonomic diversity.

### Data Analysis

Statistical analyses were performed in R Studio (R Core Team, 2024). The RFU values were first cleaned of extreme outliers (i.e., several extremely high values due to contamination). Algal growth rates were calculated from measured RFU over time using an exponential growth model. In cases where clear exponential growth was not observed, growth rates were not calculated, and the raw fluorescence data were presented instead. One-way analysis of variance or independent two-sample *t* tests were used to assess significant differences between different treatments. Data visualization was performed using R (version 4.3.1) and the ggplot2 package.

## Results and Discussion

### Stimulating Effect of Deep-Sea Mining Slurry and Sediments on Phytoplankton Growth

The data demonstrate that both deep-sea mining slurry and suspended sediment particles significantly stimulated the growth of the studied phytoplankton. Specifically, the diatom *T. weissflogii* exhibited a markedly higher exponential growth rate in the presence of slurry compared to that without slurry (0.40 d^–1^ vs 0.01 d^–1^, *p* < 0.01; Figure 1). The stimulating effect probably resulted from the substantial release of essential nutrients and metals, and their concentrations in the slurry were 36.9 μM nitrate, and 0.7 μM phosphate with dissolved metal concentrations of Fe, Zn, Ni, Cu, Cd, Pb, Mn, and Co being 1.1, 13, 220, 21, 2.3, 0.001, 17, and 0.05 nmol L^–1^, respectively. In real-world polluted seawater, the stimulating effect on phytoplankton growth may be similar to or differ from the effect observed here, depending on the wide range of possible dilution factors between slurry and ambient seawater. A previous study indicated that deep-sea sediment disturbances release silicic acid, dissolved organic nitrogen, and metals but do not significantly increase phosphate and nitrate levels.^32^

**Figure 1:**
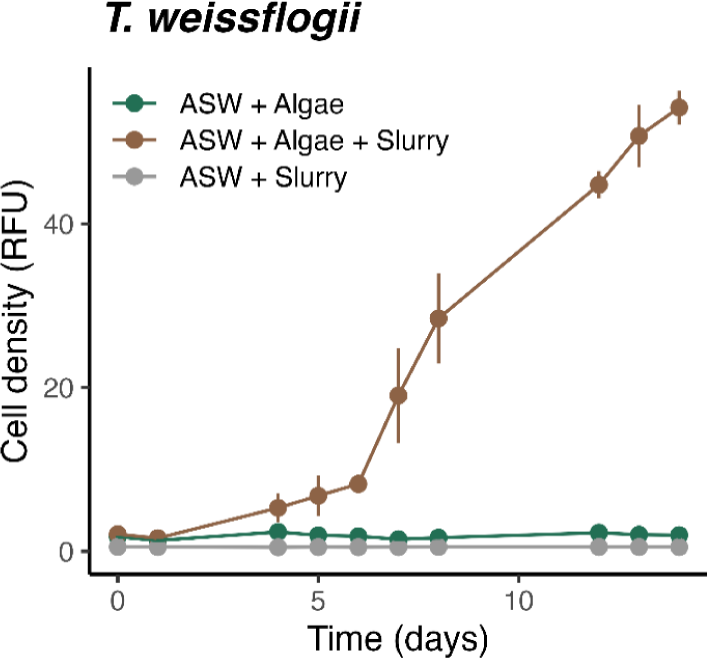
Growth of marine diatom *Thalassiosira weissflogii* in the absence or presence of deep-sea mining slurry (volume ratio of artificial seawater ASW to nodule slurry = 1:1). The artificial seawater was pretreated with Chelex-100 resin to remove background metals. The slurry was collected as it was falling off the ship’s deck during a minor accidental spill from a deep-sea mining test in the Clarion–Clipperton zone. Data points represent means ± standard deviation, the exponential growth rate in the presence of slurry was significantly higher than that without slurry (0.40 d^–1^ vs 0.01 d^–1^, *p* < 0.01). The slurry alone contributed to a negligible relative fluorescence unit of 0.53 ± 0.01 RFU (*n* = 20).

The stimulatory effect was also observed in *T. weissflogii* and *S. costatum* when exposed to Clarion–Clipperton zone sediments in natural Pacific seawater, particularly at higher concentrations (Figure 2). Specifically, the exponential growth rates of *T. weissflogii* were 0.02 d^–1^ at 10 mg L^–1^, 0.15 d^–1^ at 20 mg L^–1^, and 0.14 d^–1^ at 50 mg L^–1^, and the rates for *S. costatum* were 0.01 d^–1^ at 10 mg L^–1^, 0.04 d^–1^ at 20 mg L^–1^, and 0.10 d^–1^ at 50 mg L^–1^, which were significantly higher than 0 d^–1^ in the control (*p* < 0.05), except that *p* = 0.06 for *T. weissflogii* at 50 mg L^–1^. Notably, there was an initial slight decrease in cell density of *T. weissflogii*, and the growth enhancement of both diatoms became more apparent after 20 days of exposure, suggesting a delayed biological response. This unexpected delay could be attributed to the initial complexation of released metals by strong organic ligands in natural seawater,^33^ rendering them bioavailable only after induction of high-affinity uptake systems in phytoplankton.^34^ No growth was observed in the absence of Clarion–Clipperton zone sediments due to the low background concentrations of nutrients and metals in the natural seawater sample (see Materials and Methods). Such limited growth of the diatom *S. costatum* was also observed when they were exposed to 2, 5, or 10 mg L^–1^ suspended sediment particles.

**Figure 2:**
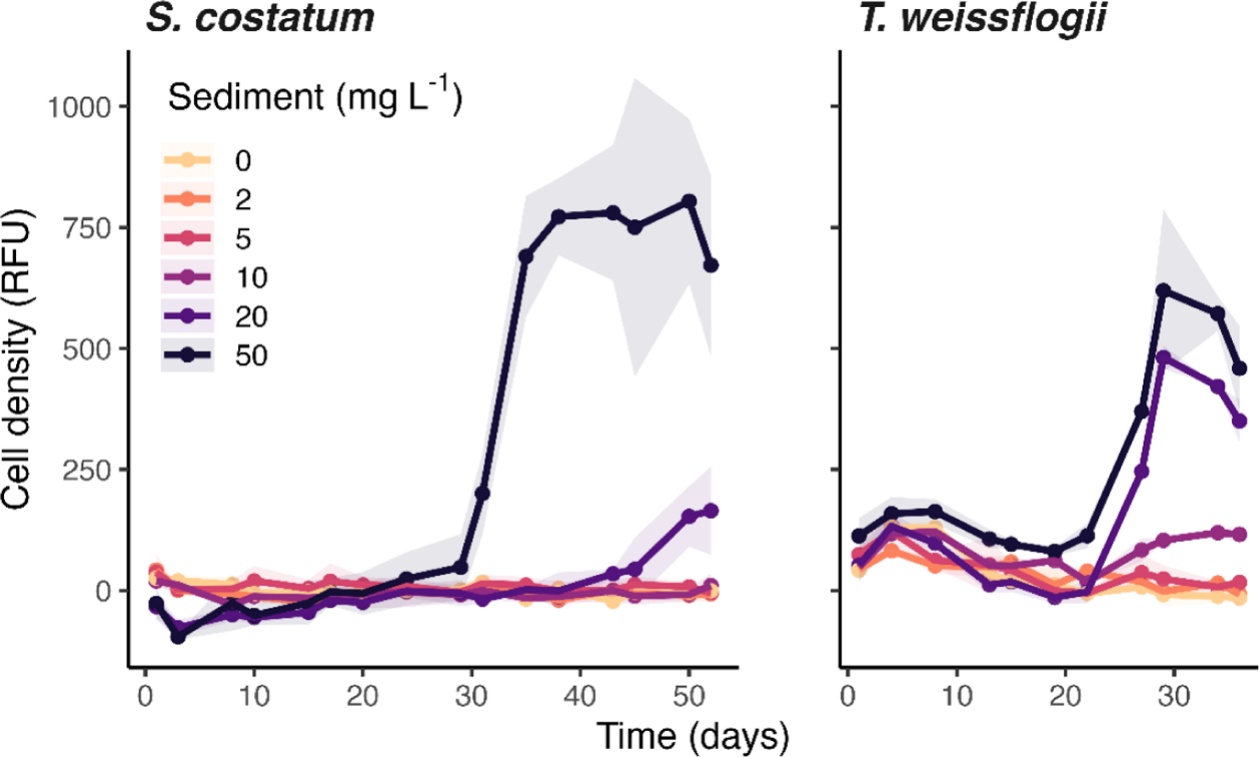
Growth of marine diatoms *Thalassiosira weissflogii* and *Skeletonema costatum* in the presence of 0 to 50 mg L^–1^ suspended sediment particles in surface seawater from the Pacific Ocean. Data points with shading represent means ± standard deviation. The exponential growth rates of *T. weissflogii* were 0 d^–1^ at 0, 2, and 5 mg L^–1^, 0.02 d^–1^ at 10 mg L^–1^, 0.15 d^–1^ at 20 mg L^–1^ and 0.14 d^–1^ at 50 mg L^–1^; the rates of *S. costatum* were 0 d^–1^ at 0 mg L^–1^, 0.01 d^–1^ at 2 mg L^–1^, 0 at 5 mg L^–1^, 0.01 d^–1^ at 10 mg L^–1^, 0.04 d^–1^ at 20 mg L^–1^ and 0.10 d^–1^ at 50 mg L^–1^. The sediment was collected from the 0–0.5 cm of the Clarion–Clipperton zone. No additional nutrients were supplied to the natural seawater. Background relative fluorescence unit (RFU) signals from the sediment particles were subtracted (Figure S2).

The stimulating effect of Clarion–Clipperton zone sediment particles extended to three other phytoplankton species: *E. huxleyi*, *P. tricornutum*, and *Synechococcus* sp. Their exponential growth rates in the presence of 30 mg L^–1^ sediment particles were 0.33 d^–1^, 0.58 d^–1^, and 0.02 d^–1^, respectively, significantly higher than in artificial seawater without added sediments (*p* < 0.01; Figure S3). Unlike the delayed response in Pacific Ocean seawater, *T. weissflogii* exhibited enhanced growth within 2 days in artificial seawater, suggesting that metals from the sediment particles were more readily bioavailable in artificial seawater. However, interspecies differences were evident, as the cyanobacterium *Synechococcus* showed only a minor growth increase (0.02 d^–1^).

The differing responses of phytoplankton species to deep-sea mining sediments likely reflect variations in their elemental stoichiometry,^39^ trace metal requirements,^35,36,40^ and physiological tolerance to metal exposure. For instance, diatoms such as *T. weissflogii* and *P. tricornutum* typically have high Fe and Si demands and possess efficient uptake systems that may enable them to benefit more from sediment-released nutrients and metals. In contrast, *Synechococcus sp.*, which generally thrives in oligotrophic waters with low nutrient and low metal availability, may be more susceptible to metal toxicity—particularly from elevated Cu concentrations.^37,38^ These interspecies differences suggest that deep-sea sediment discharges could selectively favor certain phytoplankton groups over others, leading to shifts in community composition. Such changes have important ecological implications, as they can influence primary productivity, nutrient cycling, and trophic transfer efficiency in the surface ocean. Our findings highlight the need to consider species-specific traits when evaluating the potential ecosystem-level impacts of deep-sea mining activities.

### Availability of Nitrogen and Metals from Deep-Sea Mining Region Sediments to Phytoplankton

The study confirmed that Clarion–Clipperton zone sediments release both nitrogen and bioavailable trace metals that are essential for phytoplankton growth. In artificial seawater with sufficient trace metals but no added nitrogen, the presence of 30 mg L^–1^ suspended Clarion–Clipperton zone sediment particles significantly increased both maximal cell yield and exponential growth rates of *E. huxleyi* (0.39 d^–1^), *T. weissflogii* (0.53 d^–1^), and *P. tricornutum* (0.57 d^–1^) compared to their controls without sediment (*p* < 0.01, Figure S4). Similarly, in nitrate-sufficient but metal-free artificial seawater, the presence of Clarion–Clipperton zone sediments led to significant growth increases, with *E. huxleyi*, *T. weissflogii* and *P. tricornutum* reaching growth rate of 0.39 d^–1^, 0.67 d^–1^ and 0.77 d^–1^, respectively, which were significantly higher than the controls (*p* < 0.01, Figure S4). The presence of the sediment particles also increased the cell yield of *Synechococcus sp.*, but no significant growth was observed. This suggests that if deep-sea mining discharge is spilled into surface waters, it could impact primary producers in oligotrophic ocean regions where nitrogen and/or trace metals are limiting,^15^ such as those over the CCZ.

Trace metals had a more pronounced impact on phytoplankton growth than nitrogen. The exponential growth rates and maximal cell yields were generally higher in metal-free artificial seawater with added sediment than in nitrogen-free artificial seawater with sediment (Figure S4), likely due to the lower absolute metal requirements of algae and limited release of nitrogen from the sediment. These findings align with previous research showing significant metal release but minimal nitrate release from deep-sea sediments.^32^ However, *Synechococcus* exhibited little growth, again suggesting a potential toxic effect from sediment-released metals.

Further investigation revealed different bioavailability of metals in the Clarion–Clipperton zone sediment (Figure 3). Removing individual metals from the exposure medium resulted in the growth cease of *T. weissflogii* and *S. costatum*. The presence of sediment particles significantly enhanced the exponential growth of both diatoms in the medium with individual metal exclusions (*p* < 0.01). However, the maximal cell yield did not reach the levels observed in the positive control, particularly for *T. weissflogii*. The maximal cell yields of *T. weissflogii* followed the order of Cu > Mn > Co > Ni > Zn > Fe exclusion in sediment added experiment, suggesting that Cu in the sediment particles almost satisfied their growth requirement while Fe was insufficient. However, this does not necessarily indicate that Cu had the highest bioavailable concentration in the sediment, as the growth requirement for each metal varies.^39^ The enhanced growth would be primarily due to the target metal being available in the sediment, rather than those nontargeted metals, because the latter were already present in sufficient concentrations to support algal growth (Table S2).

**Figure 3:**
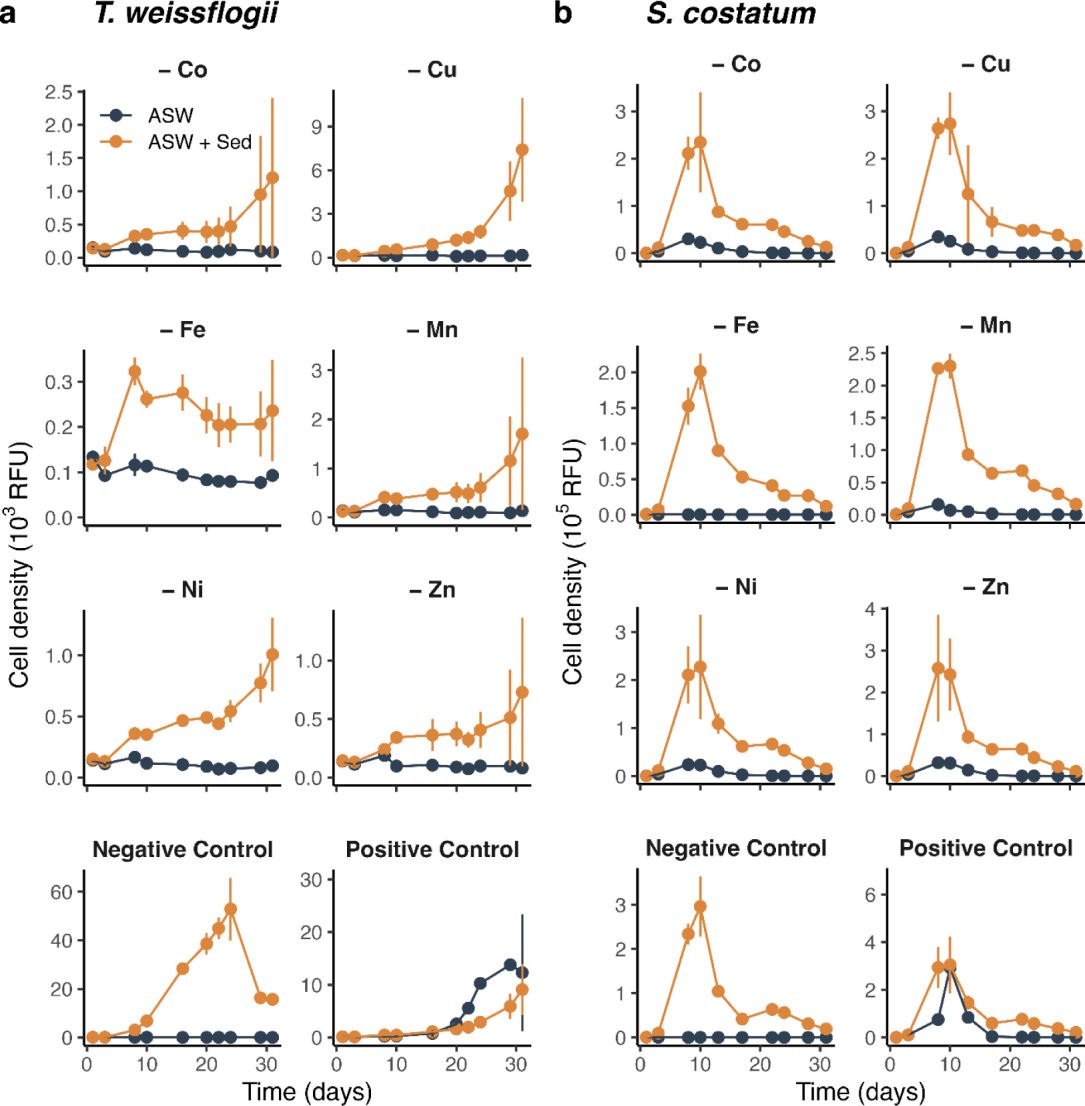
Growth of marine diatoms *Thalassiosira weissflogii* and *Skeletonema costatum* in the absence or presence of ∼6 mg L^–1^ suspended sediment particles in ASW. The ASW was supplied with sufficient chelex-100 resins pretreated N, P and Si but one essential metal was excluded. Negative Control means no metals were added, while Positive Control includes all essential metals. Data points represent means ± standard deviation. The sediment was collected from the 0–0.5 cm of the Clarion–Clipperton zone.

While our bioassay approach does not directly quantify metal speciation or bioavailability, it provides a biologically relevant assessment of whether sediment-derived metals are accessible to phytoplankton under limiting conditions. Differences in cell yield and growth rate relative to positive (sufficient metals) and negative controls (no targeted metal) allow us to infer both the availability and sufficiency of the targeted metals. Direct measurements of metal speciation in sediments or porewaters would offer further insights, though linking speciation to bioavailability remains complex due to the diverse and dynamic metal uptake strategies of marine phytoplankton.^41^ At present, determining metal speciation on low volume porewater samples from sediments remains a challenge analytically. Sequential chemical extraction of the CCZ sediment showed that 29% of the Fe (1.14 wt % Fe_ox_ relative to 4.42 wt % total Fe) in sediments were present as authigenic reactive Fe oxyhydroxide minerals, which are known to adsorb other metals to their surface. Similarly, Mn oxides are also present and also form strong surface-adsorbed metal complexes with Cu, Co, Ni and Zn. We anticipate these authigenic and reactive Fe oxyhydroxide minerals are in general more bioavailable than the more crystalline and less reactive silicate phases, potentially driving the growth observed in experiments, however, the bioavailability of reactive Fe minerals has also been shown to vary between different phytoplankton,^42^ and any distinctions between metals adsorbed to Mn oxide and Fe oxyhydroxide have not yet been made.

There was likely a toxic effect of Clarion–Clipperton zone sediment on *T. weissflogii* under metal-replete conditions, as indicated by the following observations. First, the exponential growth of *T. weissflogii* in the positive control (i.e., with sufficient metals) was significantly lower when Clarion–Clipperton zone sediment was added compared to when it was not (0.14 d^–1^ versus 0.20 d^–1^, *p* < 0.01, Figure 3). This phenomenon was further confirmed by an independent experiment (Figure S5). Second, the exponential growth in the negative control (i.e., without added metals) with sediment was markedly higher than in the positive control with sediment (0.42 d^–1^ versus 0.14 d^–1^, *p* < 0.01), suggesting that excess metals in the latter had an inhibitory effect. Given that the concentrations of N, P, and Si were maintained at levels sufficient to sustain phytoplankton growth (Table S2), nutrient limitation can be excluded as the primary cause of the observed growth reduction. However, the same sediment exhibited a stimulatory effect on *T. weissflogii* in the Pacific Ocean seawater (Figure 2). One possible explanation for this contrasting effect is the difference in metal bioavailability, influenced by metal concentration, speciation and the concentration of metal-chelating ligands present. A study^43^ concluded that deep-sea mining activities are unlikely to release toxic Cu^2+^ concentrations into seawater, as more than 99% of Cu was organically complexed. However, in high-UV open ocean regions, photoreduction may enhance metal reactivity and bioavailability,^44^ increasing the risk of Cu toxicity should any discharge or leaks occur from mining ships. Other contributing factors may include sediment-associated organic toxins, alterations in seawater chemistry, and interactions with microbial communities. Further investigation into these aspects is needed to improve predictions of the availability and ecological effects of deep-sea mining waste on phytoplankton.

### Inhibition of Copepod Growth and Reproduction by Deep-Sea Mining Region Sediments

Unlike phytoplankton, the growth and reproduction of the marine copepod *T. californicus* were inhibited by the presence of Clarion–Clipperton zone sediment particles, with higher particle concentrations leading to increased mortality (Figure 4). After 26 days of exposure, the number of surviving copepods declined from an initial 20 individuals to 12 ± 0.5 (2 mg L^–1^ particles), 11 ± 1.4 (5 mg L^–1^), 9 ± 1.3 (20 mg L^–1^), and only 2 ± 1.6 (50 mg L^–1^), which were all significantly lower than 50 ± 10.6 in the positive control (*p* < 0.01). Similarly, exposure to North Pacific abyssal sediment significantly reduced copepod growth and reproduction compared to the control (Figure 5). Specifically, following the exposure to 2 to 20 mg L^–1^, the number of mating pairs (0), pregnant individuals (0), and newborns (0 to 4 ± 4.3) were all markedly lower in the presence of sediment particles than the Positive Control (4 ± 1.4 mating pairs, 3 ± 0.8 pregnant individuals and 28 ± 7.5 newborns, respectively, *p* < 0.01 for all). The reduced reproduction and higher mortality were likely due to particle ingestion, their poor nutritional quality, reduced food ingestion, and the toxic effects of metals released from the sediment. Our results correspond well with studies on other copepod species. For instance, a study^19^ shows that high concentrations of small sediment particles (9.3 μm in diameter) have negative impacts on the ingestion rate, egg production and mortality of *Acartia tonsa*. A separate study^45^ found that exposure to 0–200 mg L^–1^ of artificial particles caused *A. tonsa* to exhibit increasingly erratic and slower swimming trajectories as particle concentration increased. The underlying mechanisms of the observed adverse effect of deep-sea sediment on copepods merit further investigations.

**Figure 4:**
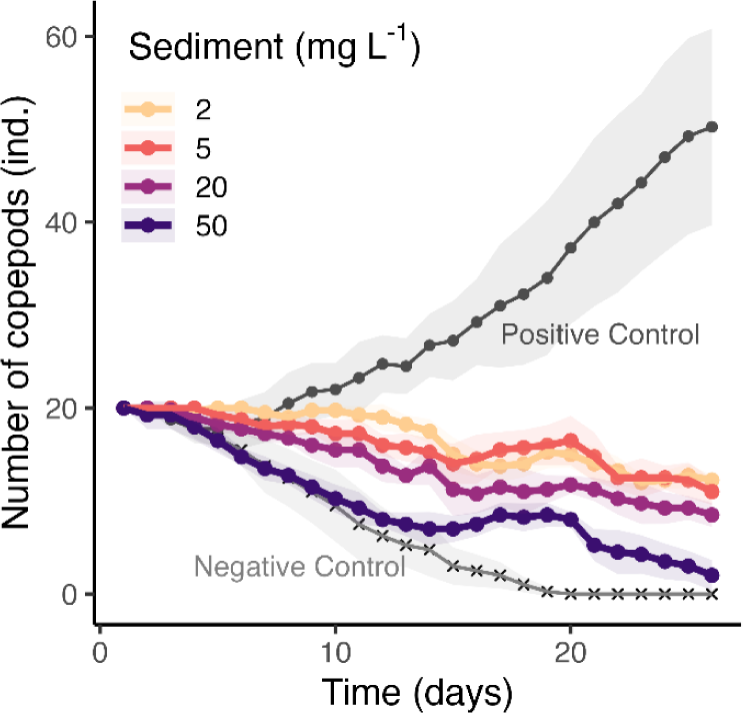
Reproduction of marine copepods *Tigriopus californicus* in the presence of 0 to 50 mg L^–1^ suspended sediment particles in ASW. The Negative Control represents copepods without any algae food supply, while the Positive Control includes algae food without exposure to sediment particles. Data points with shadows represent means ± standard deviation, the slopes were −0.38 at 2 mg L^–1^, −0.33 at 5 mg L^–1^, −0.48 at 20 mg L^–1^, −0.66 at 50 mg L^–1^, and −0.93 mg L^–1^ for the Negative Control, which were all significantly smaller than the slope of the Positive Control (i.e., 1.31, *p* < 0.01). The sediment was collected from the 0–0.5 cm of the Clarion–Clipperton zone.

**Figure 5:**
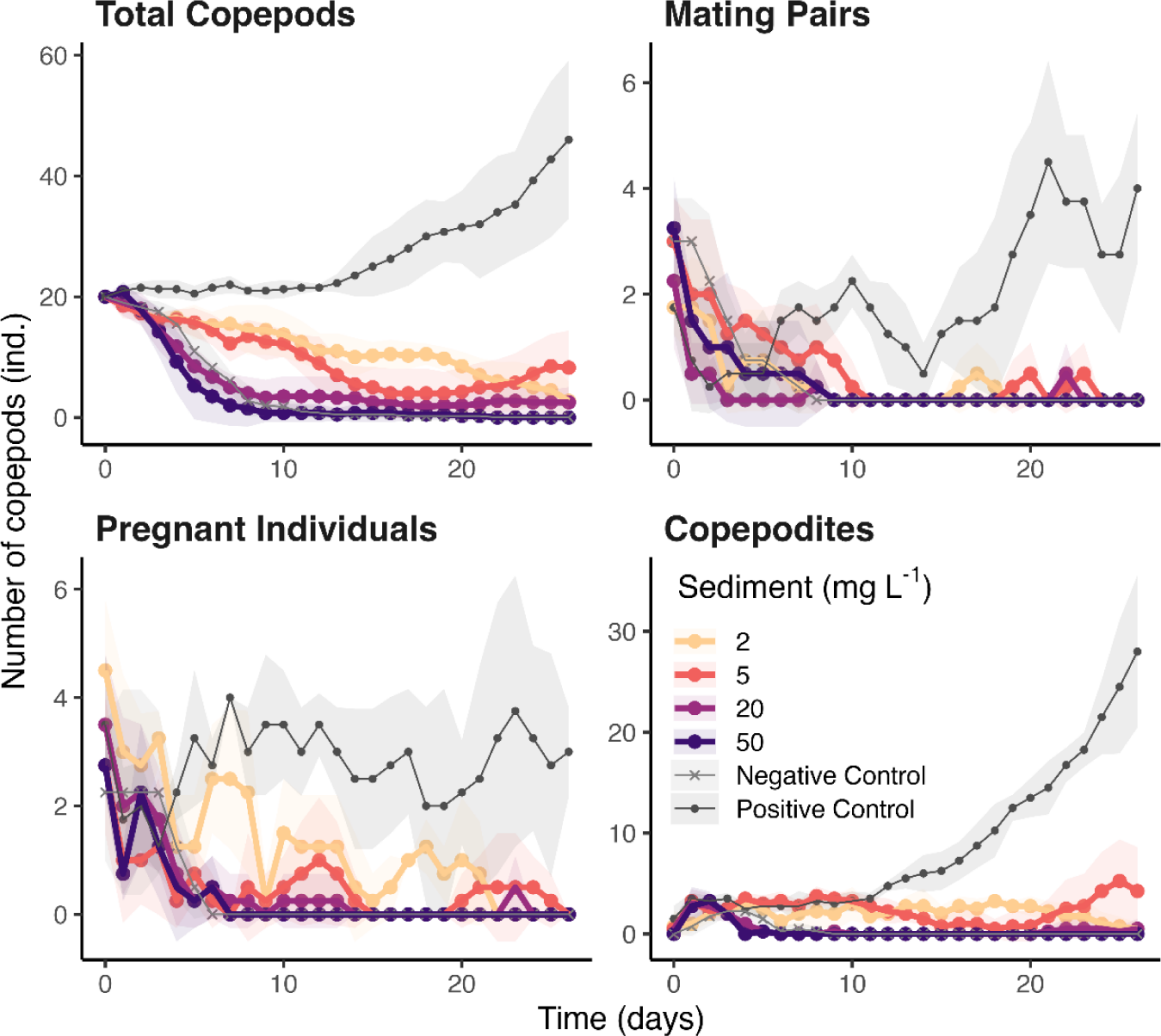
Reproduction, mating success, pregnancy rates, and offsprings of marine copepods *Tigriopus californicus* in the presence of 0 to 50 mg L^–1^ suspended sediment particles in ASW. The Negative Control represents copepods without any algae food supply, while the Positive Control includes algae food without exposure to sediment particles. Data points with shadows represent means ± standard deviation. The sediment was collected from the 0–1 cm of the North Pacific abyss.

Although most copepods (e.g., *Calanus, Acartia, Temora*) inhabit the epipelagic zone (0–200 m), some deep-water species (e.g., *Pleuromamma, Metridia, Gaussia, Spinocalanus*) migrate into or permanently reside in the mesopelagic (200–1000 m) and deeper layers (1000–6000 m),^46,47^ where deep-sea mining discharges likely happen. Collectively, these findings suggest that deep-sea mining waste discharge, either accidental or planned, could potentially reduce copepod populations, with the risk of cascading effects on higher trophic levels and hence broader ecosystem imbalances.

### Ecological Implications, Limitations of the Present Study, and Recommendations for the Future

These findings have several ecological implications. The enhanced growth of phytoplankton observed in our experiments, although suggesting nutrient enrichment, could lead to unintended consequences such as localized eutrophication and shifts in phytoplankton community structure. Such changes may alter food web dynamics and disrupt biogeochemical processes in surface waters. In contrast, the limited response of cyanobacteria *Synechococcus*, the reduced growth of the diatom *T. weissflogii* in metal-replete seawater, as well as the reduced growth and reproduction of copepods *T. californicus* following sediment exposure raises concerns about potential negative impacts on certain primary producers and higher trophic levels. Copepods play a central role in pelagic ecosystems as grazers of phytoplankton and as prey for fish and other marine organisms.^20^ A decline in copepod populations may therefore reduce food availability for key species, including commercially important fish. Additionally, the release of metals from deep-sea sediments into surface waters poses a possible risk of bioaccumulation and biomagnification through marine food webs, which may have implications for seafood safety and human health. Overall, our study highlights the ecological vulnerability of pelagic ecosystems to deep-sea mining waste discharge.

Future research should integrate field validation and ecosystem modeling to better predict the long-term consequences of deep-sea mining discharge activities. Several limitations of the present study should be acknowledged.

Lack of *in situ* conditions: Our experiments were conducted under controlled laboratory conditions, which may not fully capture the complexity of *in situ* physical, chemical and biological conditions. In the natural environment, multiple factors such as hydrodynamic mixing, microbial interactions, variable light conditions, and oxygen minimum zones^48^ could influence the bioavailability and effects of slurry and/or sediment-released nutrients and metals. The interplay between these factors could lead to different responses from those observed in the laboratory.
Simplified metal speciation analysis: Our study focused on the bioavailability of metals but did not fully account for the complex chemical transformation of metals in the ocean. In natural seawater, organic ligands, photochemical reactions, and microbial activity can alter metal speciation, which in turn affects their bioavailability and potential toxicity to marine organisms. For example, recent *in situ* observations showed that sediment resuspension decreases trace metal inventories because of metal scavenging,^49^ highlighting the dual role of sediment particles as a sink and a source of dissolved metals. More detailed speciation analyses under *in situ* conditions are required to improve the ecological relevance of these findings.
Short-term exposure studies: The duration of the experiments was relatively short compared to the time scales over which deep-sea mining discharge plumes may persist and keep changing. It is possible that mining vessels will occupy and/or reoccupy a site for weeks to years. Long-term exposure could result in different biological responses due to cumulative effects, potential adaptation mechanisms, or shifts in community composition. Further studies incorporating long-term monitoring are necessary to assess the chronic effects of deep-sea mining discharge.
Limited representation of marine communities: The phytoplankton and copepod species used in this study are native to surface and near-surface waters and may not directly represent the taxa most affected by deep-sea mining discharge if it occurs at greater depths. However, these species were selected as ecologically relevant model organisms with well-established laboratory protocols, allowing controlled mechanistic exploration of sediment-driven nutrient enrichment and metal toxicity. The responses observed—nutrient-stimulated primary production, metal-induced growth inhibition, and reproductive disruption—represent processes likely to occur across a range of pelagic species, including those inhabiting mesopelagic or deeper layers. Therefore, while specific sensitivities may vary, the mechanisms of impact demonstrated here are pertinent to evaluating potential risks across pelagic ecosystems. Future research using species native to greater depths and under *in situ* conditions would further refine impact predictions.

As member states are negotiating the Mining Code at the ISA and options to protect the marine ecosystem from deep-sea mining, recent research documents long-term disruptions to groups of benthic organisms from test mining experiments conducted four decades ago.^12^ However, the knowledge regarding impacts across pelagic zones, particularly where the mining discharges are expected, remains limited. We provide a first of its kind research in this area, highlighting ecological vulnerabilities of pelagic ecosystems when exposed to potential deep-sea mining waste discharges. Our findings strengthen the necessity for further research to examine the interconnectivity and ecological consequences of potential deep-sea mining disturbances coupling benthic and pelagic realms. Such integrated and holistic scientific evidence is essential to ensure effective protection of both the deep-sea and the broader marine ecosystems.

## Supplementary Material





## Data Availability

The data sets analyzed during the current study are open accessible in the Figshare repository via the DOI link 10.6084/m9.figshare.28931729.

## Notes

Methods of CCZ sediment analysis; chemical properties of deep-sea mining slurry, deep-sea sediments, and artificial seawater; effect of sediment particles on the background signals of RFU; growth of phytoplankton *E. huxleyi*, *T. weissflogii*, *P. tricornutum*, and *Synechococcus* sp. in the absence or presence of sediment particles in either N-free or metal-free artificial seawater; an independent experiment confirming that the difference in cell yield of *T. weissflogii* exposed to the sediment in the positive and negative controls (PDF)
All data reported in the present study (XLSX)
