# Correction to “Ecological Impacts of Deep-Sea Mining Waste on Marine Algae and Copepod *Tigriopus californicus*”

**DOI:** 10.1021/acs.est.6c01484

**Published:** 2026-02-11

**Authors:** Catherine Thomson, Alastair J. M. Lough, Jean Moorkens, Te Liu, Shelby A. Gunnells, Jessica N. Fitzsimmons, Zvi Steiner, Ann G. Dunlea, Clare Woulds, William B. Homoky, Mengjiao Wang, Qiao-Guo Tan, Fengjie Liu

The authors thank the captain, crew, and scientific team of the SO289 research expedition and Te Liu, Zhongwei Yuan, and Kechen Zhu at GEOMAR for their help in collecting and shipping the seawater. The research cruise was funded by Bundesministerium für Bildung and Forschung (BMBF) Grant ‘South Pacific GEOTRACES’ (Förderkennzeichen 03G0289NA).

