# Supplementary material for: Ecological Impacts of Deep-Sea Mining Waste on Marine Algae and Copepod Tigriopus californicus
Source: Environ Sci Technol. 2025 Sep 18;59(38):20190–200. doi: 10.1021/acs.est.5c06113 (PMC12490012; doi:10.1021/acs.est.5c06113)
Supplement: Supplementary file 1 [file es5c06113_si_001.pdf]

Supporting Information

Ecological Impacts of Deep-Sea Mining Waste on Marine Algae and Copepod *Tigriopus californicus*

[1]Catherine Thomson, [2]Alastair J.M. Lough, [3]Jean Moorkens, [4]Te Liu, [5]Shelby A. Gunnells, [5]Jessica N. Fitzsimmons, [6]Zvi Steiner, [7]Ann G. Dunlea, [2]Clare Woulds, [2]William B. Homoky, [8]Mengjiao Wang, [9]Qiao-Guo Tan, [3,10]Fengjie Liu*

1 Centre for Environmental Policy, Imperial College London, Exhibition Road, London, SW7 2AZ, UK

2 Faculty of Environment, University of Leeds, Leeds, LS2 9JT, UK

3 Department of Life Sciences, Imperial College London, Silwood Park, Buckhurst Rd, Berks, SL5 7PY, UK

4 School of Ocean and Earth Science, University of Southampton, Southampton, SO14 3ZH, UK

5 Department of Oceanography, Texas A&M University, College Station, TX 77843, USA

6 Fredy and Nadine Herrmann Institute of Earth Science, The Hebrew University of Jerusalem, Givat Ram, Jerusalem, 9190401, Israel

7 Department of Marine Chemistry and Geochemistry, Woods Hole Oceanographic Institution, Woods Hole, MA 02543, USA

8 Greenpeace Research Laboratories, School of Bioscience, University of Exeter, Exeter, EX4 4RN, UK

9 Fujian Provincial Key Laboratory for Coastal Ecology and Environmental Studies, State Key Lab of Marine Environmental Science, College of the Environment and Ecology, Xiamen University, Xiamen, Fujian, 361102, China

10 Grantham Institute - Climate Change and the Environment, Imperial College London, Exhibition Road, London, SW7 2AZ, UK

*Corresponding author: fengjie.liu@imperial.ac.uk

This file has 10 pages, including 1 note, 5 figures and 2 tables.

Methods of CCZ sediment analysis

For TOC and TN a 0.4g sample (0.1 g for reference materials that contained more carbon) from the original sample bag was weighed into a 30 mL universal tubes, which was then filled to the 25mL line with 2M HCl. The sub-sampled acid solution was oven heated at 60 °C for 2 hours and left overnight to allow all carbonate to be removed as CO_2. The samples were then centrifuged and the acid decanted. The samples were then washed twice using distilled water and centrifugation. After acid washing, the samples were oven dried at 60 °C. After drying, the samples were re-ground in-situ. Samples were analysed using an Elemental Analyser - Isotope Ratio Mass Spectrometry (EA-IRMS, Europa Scientific). Sample preparation and analysis was done by *sercon analytical, UK*. The typical RSD on repeat measurements of samples was 2 % for TOC content and 0.7 % for TN. Sediment porosity was determined from the difference between the wet and dry weight. Particle size was determined by resuspending 3 ml of wet sediment in 50 ml 5 % sodium hexametaphosphate (*Calgon)* solution, placed on a shaker table for 2 hours at 95 rpm then analyzed using a Beckman Coulter LS230. Total metal concentrations in sediment were determined by dissolution in 5 ml concentrated HNO_3 with 2 ml of concentrated hydrofluoric acid (HF) and 1 ml perchloric acid ($HClO_4$). Sample-acid solutions were heated overnight at 70 °C on a hot plate under a fume hood equipped with a scrubber. The acid solutions were then evaporated on the hot-plate and the heating-drying procedure was repeated with 2 ml of boric acid (H_3BO_3). Dry samples were re-dissolved in 50 % HNO_3 and placed back on the hot plate until they fully dissolved. This solution was diluted to 5 % HNO_3 for analysis by ICP-MS (*iCAPQc, thermoscientific*). Repeat analysis of certified reference sediments (PACS-1, SBC-1, SGR-1B) gave an RSD of 16 % on average (across all elements and reference standards). On average measured element concentrations for reference standards were 98 ± 9 % of certified values for PACS-1, 104 ± 10 % for SBC-1 and 102 for SGR-1B ± 15 %. Fe mineralogy was examined using a sequential extraction [1]. A sodium acetate extraction was used to assess the concentration of Fe associated with carbonate minerals. A sodium dithionite extraction was used to assess the concentration of Fe oxide minerals and an ammonium oxalate/oxalic acid extraction to assess the concentration of mixed oxidation state minerals such as magnetite. Extraction solutions are dried down and re-dissolved in dilute nitric acid for analysis of Fe concentration using atomic absorption spectroscopy (AAS, iCE 3000 series*, thermoscientific*). Measured values of the WHIT reference standard were within 13 % or less of consensus values for all Fe fractions. Repeat analysis of the WHIT standard gave an RSD of 6 % for Fe_{carb}, 16 % for Fe_{ox} and 11 % for Fe_{mag}.

Table S1. Chemical properties of deep-sea mining slurry and deep-sea sediments used in the present study.

Sample	Coordinates	N	P	Al	Cd	Co	Cr
Clarion-Clipperton Zone sediment[1]	10°50′24″N, 116°09′00″W	-	-	-	-	90 mg/kg	-
North Pacific abyssal plain sediment[2]	49°50.4′N, 149°37.7′W	-	0.09%	6.99%	-	27mg/kg	25mg/kg
Deep-sea mining slurry	10°19'43"N, 117°11'6"W	36.9µM	0.7µM		2.3nmol/kg	0.05 nmol/kg	
	Cu	Fe	Mn	Mo	Ni	Pb	Zn
Clarion-Clipperton Zone sediment[1]	600mg/kg	4%	1%	-	400mg/kg	-	200mg/kg
North Pacific abyssal plain sediment[2]	125mg/kg	5.45%	0.16%	1mg/kg	22mg/kg	14mg/kg	95mg/kg
Deep-sea mining slurry	21 nmol/kg	1.1 nmol/kg	17 nmol/kg		220nmol/kg	0.60 pmol/kg	13nmol/kg

Note, 1 the metal concentration is based on wet weight of the sediment.

2 the metal concentration is based on dry weight of the sediment, for other details see the dataset[2].

Table S2. Concentration of major salts, major nutrients, trace metals and vitamins in the artificial growth medium.

**Artificial Seawater (ASW)**	**Concentration (mol L^{-1})**
NaCl	4.20×10^{-1}
KCl	1.01×10^{-2}
$NaHCO_3$	2.50×10^{-3}
H_3BO_3	5.82×10^{-4}
KBr	9.66×10^{-4}
NaF	7.14×10^{-5}
$MgSO_4$	2.50×10^{-2}
$MgCl_2$	2.00×10^{-2}
$CaCl_2$	1.00×10^{-2}
$SrCl_2$	6.38×10^{-5}
**Major nutrients**	
NaH_2PO_4	1.00×10^{-5}
$NaNO_3$	1.60×10^{-4}
Na_2SiO_3	1.00×10^{-4}
**Metal nutrients**	
Na_2EDTA	2.00×10^{-5}
$FeCl_3$*	4.00×10^{-9}
$CuSO_4$	1.60×10^{-8}
$ZnSO_4$	2.00×10^{-8}
$CoCl_2$	8.00×10^{-9}
$MnCl_2$	1.80×10^{-8}
Na_2MoO_4	1.00×10^{-7}
$NiSO_4$	2.00×10^{-8}
Na_2SeO3	1.00×10^{-8}
**Vitamins**	
Thiamine HCl	2.96×10^{-7}
Biotin	2.05×10^{-9}
Cyanocobalamin	3.69×10^{-10}

Note, artificial seawater ASW means one with only major salts. The ASW and stock solutions of N, P and Si were pre-treated with Chelex-100 resins to remove background metals. In Figures 2 and 3, the growth media had 40 nM Fe but the experimental media for the metal exclusions had 4 nM Fe. In all other algae bioassays, the growth and experimental media had 4 nM Fe. 40 nM Fe was supplied in the growth media to maintain a high growth rate, whereas 4 nM Fe was used in the exposure experiments to mimic the low Fe concentrations of open-ocean surface waters.

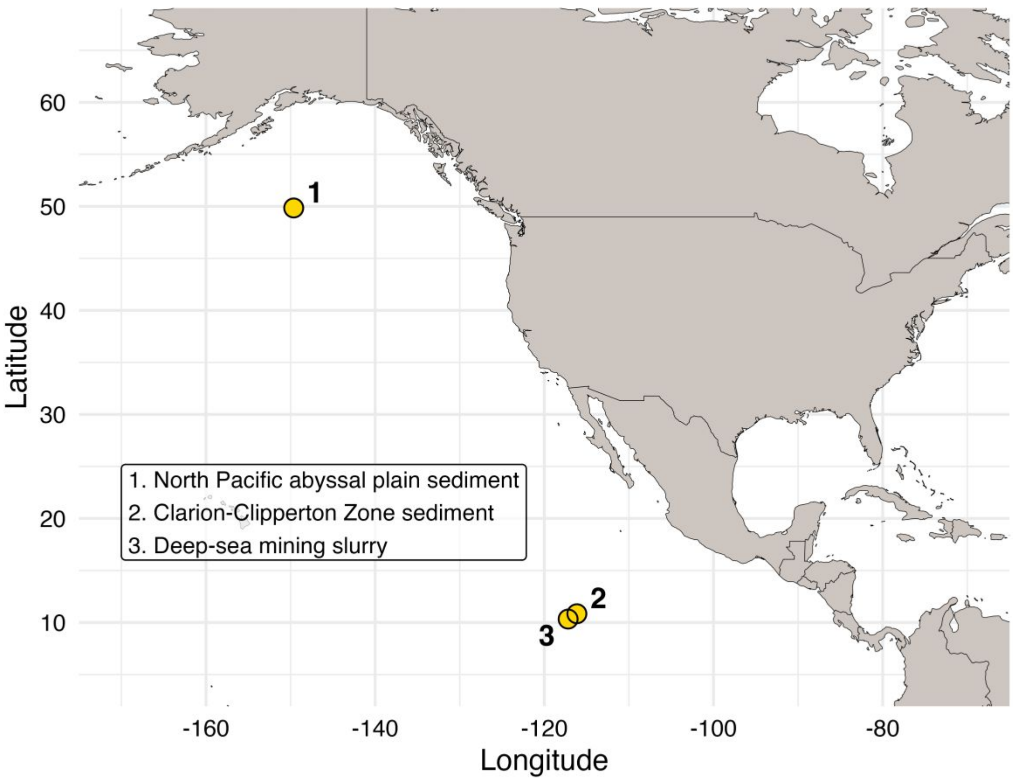

Figure S1. Geographic locations of the deep-sea mining slurry and sediment sampling sites used in this study.

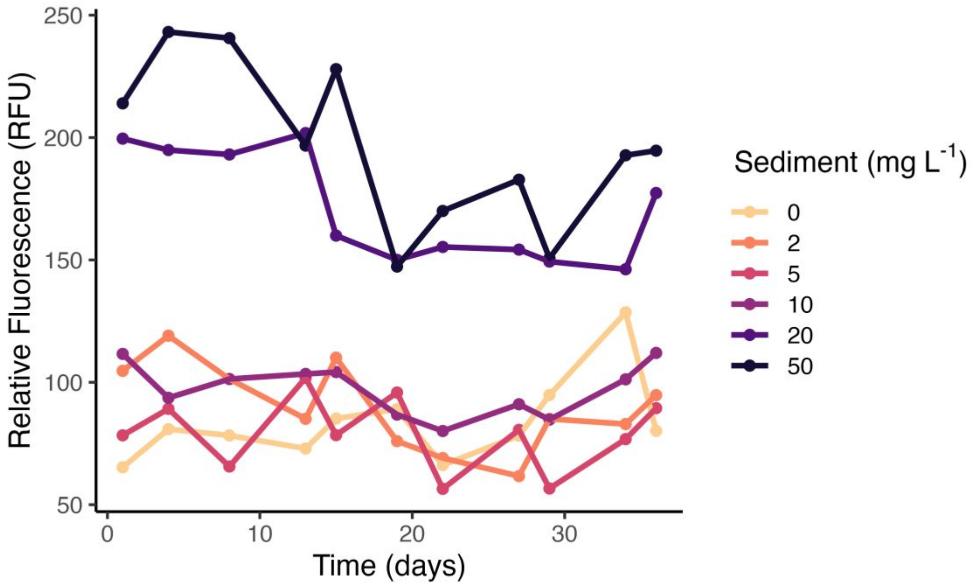

Figure S2. Effect of suspended sediment particles (0 to 50 mg L^{-1}) on the background signals of Relative Fluorescence Units (RFU) in ASW. This control experiment corresponds to Figure 2.

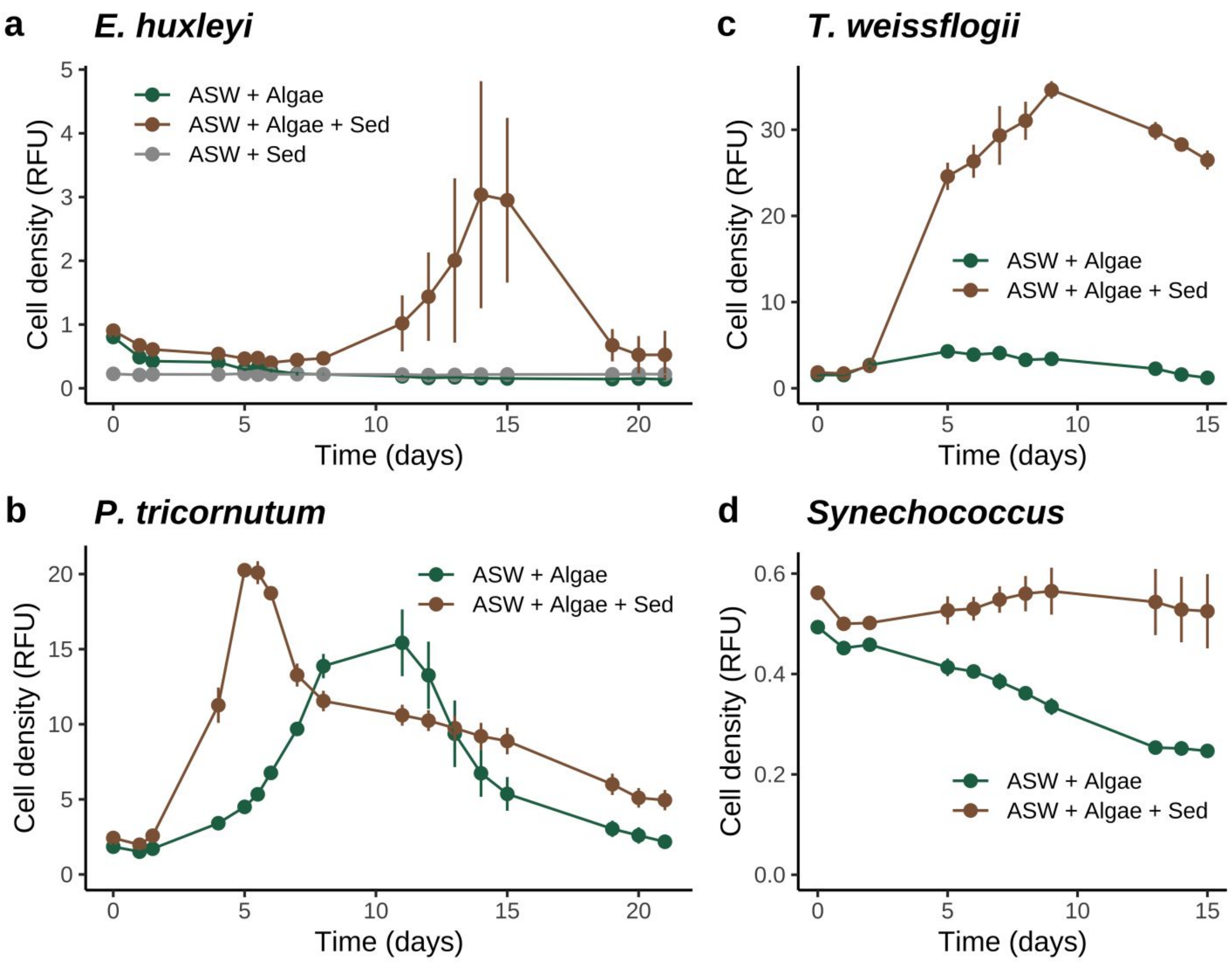

Figure S3. Growth of marine phytoplankton *Emiliania huxleyi, Thalassiosira weissflogii, Phaeodactylum tricornutum,* and *Synechococcus* sp. in the absence or presence of ~30 mg L^{-1} suspended sediment particles in ASW. Data points represent means ± standard deviation, and the exponential growth rates in the presence of the particles were 0.33 d^{-1}, 0.69 d^{-1}, 0.58 d^{-1}, and 0.02 d^{-1}, which are significantly higher than their controls ($p < 0.05$). The ASW was supplied with chelex-100 resins pre-treated P and Si. The sediment was collected from the 0-0.5 cm of the Clarion-Clipperton Zone, and the sediment particles alone contributed background signals of 0.22 ± 0.01 (n = 103, panel a).

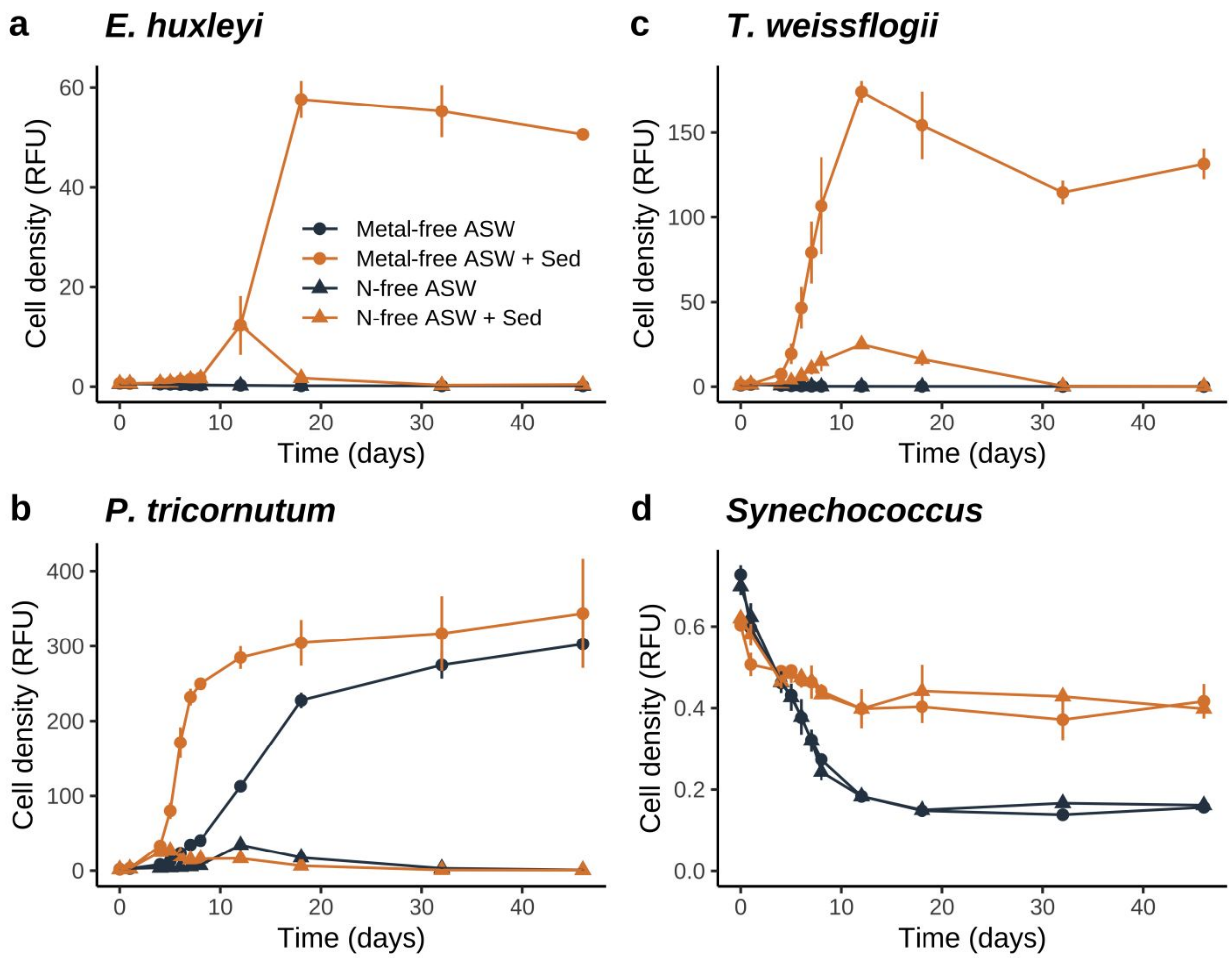

Figure S4. Growth of marine phytoplankton *Emiliania huxleyi, Thalassiosira weissflogii, Phaeodactylum tricornutum,* and *Synechococcus* sp. in the absence or presence of ~30 mg L^{-1} suspended sediment particles in either nitrogen-free or metal-free ASW. Data points represent means ± standard deviation. The ASW was supplied with chelex-100 resins pre-treated P and Si, and the sediment was collected from the 0 - 0.5 cm of the Clarion-Clipperton Zone.

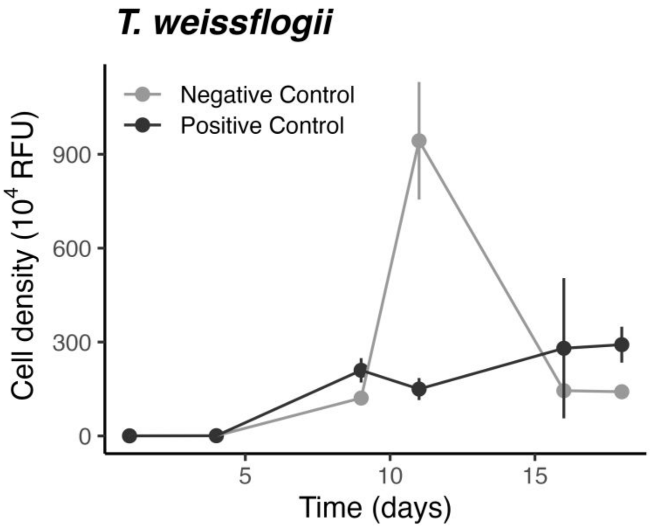

Figure S5. An independent experiment confirming that the maximal cell yield of marine diatom *Thalassiosira weissflogii* was significantly lower ($p < 0.01$) in the presence of ~6 mg L^{-1} suspended sediment particles in seawater with added metals (Positive Control) compared to that without metals (Negative Control). This suggests a toxic effect of metals released from the sediments. See Table S2 for metal and ligand concentrations. Dots represent means ± standard deviation. The ASW was supplied with sufficient chelex-100 resins pre-treated N, P and Si. Data points represent means ± standard deviation. The sediment was collected from the 0 - 0.5 cm of the Clarion-Clipperton Zone.

Reference

(1) Poulton, S. W. *The Iron Speciation Paleoredox Proxy*; Elements in Geochemical Tracers in Earth System Science; Cambridge University Press, 2021. DOI: 10.1017/9781108847148.
(2) Steiner, Z.; Antler, G.; Berelson, W. M.; Crockford, P. W.; Dunlea, A. G.; Hou, Y.; Adkins, J. F.; Turchyn, A. V.; Achterberg, E. P. Major and trace element concentrations of red clay bulk marine sediment from the North Pacific CDisK-IV expedition. PANGAEA: 2023.
